# Exclusive Enteral Nutrition Induces Remission in Pediatric Crohn's Disease via Modulation of the Gut Microbiota

**DOI:** 10.1155/2017/8102589

**Published:** 2017-10-16

**Authors:** Dawei Gong, Xinjuan Yu, Lili Wang, Lingling Kong, Xiaojie Gong, Quanjiang Dong

**Affiliations:** ^1^Department of Central Laboratories and Gastroenterology, Qingdao Municipal Hospital, School of Medicine, Qingdao University, Qingdao 266071, China; ^2^Department of Gastroenterology, The Affiliated Hospital of Qingdao University, Qingdao 266071, China; ^3^Department of Emergency Surgery, The Fifth People's Hospital of Jinan, Jinan 250022, China

## Abstract

Exclusive enteral nutrition (EEN) has been proven to be effective and safe in treating pediatric Crohn's disease (CD). EEN induces pediatric CD remission possibly through three pathways: (1) direct anti-inflammatory effects, (2) improved epithelial barrier function, and (3) modulation of the gut microbiota. Recent studies have demonstrated that modulation of the gut microbiota plays a major role in EEN-induced remission. Variations of microbial components, which directly influence the diversity and metabolic functions of the gut microbiota, are closely associated with the immunological conditions of the gut and the susceptibility to diseases. The reduction of proinflammatory microbial components and harmful microbial metabolites after EEN treatment greatly decreases the inflammatory injuries of the gut.

## 1. Introduction

Crohn's disease (CD), a major phenotype of inflammatory bowel disease (IBD), is a chronic and relapsing inflammatory process. Delayed growth and development, which is mainly caused by decreased nutrient intake, intestinal malabsorption, and protein-losing enteropathy, is commonly observed in pediatric CD patients. In recent years, exclusive enteral nutrition (EEN) treatment has been identified as an effective treatment to induce clinical remission of CD, especially in pediatric patients [1–4]. However, while EEN has been widely accepted and frequently used as a standard procedure for treating pediatric CD patients [5, 6], the underlying mechanism of EEN-induced remission remains unclear.

The intestinal microbiota plays an important role in the initiation and progression of CD [7]. When compared to healthy controls, the diversity of the intestinal microbiota of CD patients is largely decreased [8, 9]. By using principal coordinate analysis, a method commonly used to compare the microbial structures of different subjects, Sokol et al. [10] and Sabino et al. [11] have demonstrated that the microbiota of CD patients clusters separately from that of healthy controls. These findings indicate that the microbial community of CD patients is very different from that of healthy controls. Also, between CD patients and healthy individuals, the relative abundance of the intestinal microbiota is very different at various taxonomic levels. Briefly, a decrease in the Firmicutes phylum and an increase in the Proteobacteria phylum, especially Enterobacteriaceae, was observed in CD patients [10–15].

Taken together, EEN induces CD remission in pediatric patients probably through pathways related to modulation of the intestinal microbiota [16, 17]. The purpose of this review is to summarize recent findings with regard to alterations of the intestinal microbiota (based on 16S rRNA sequencing) during EEN treatment. The relationship between the gut microbiota composition and immune homeostasis is also discussed.

## 2. Alterations of the Microbiota during EEN

A good understanding of the microbiota alterations during EEN would help to illustrate the pathogenesis of IBD. Shiga et al. [18] have shown that EEN treatment could not alter the fecal microbiota bacterial load in CD patients, implying that alterations of the bacterial composition, not only the bacterial load, may play a role in inducing remission of CD. Next generation gene sequencing technology also has been applied to study the effects of EEN treatment on the whole intestinal microbial community.

Quince et al. [19] have demonstrated that the microbiota characteristics (Shannon diversity, microbiota structure, and relative abundance of various bacterial taxa) of pediatric CD patients were significantly different from those of healthy controls. Interestingly, during EEN treatment, the microbial diversity was further decreased and the community structure became more dissimilar from those of healthy controls. In addition, operational taxonomic units (OTUs), either positively or negatively correlated with fecal calprotectin, have been shown to be decreased during EEN treatment [19]. According to Lewis et al. [20], the gut microbiota composition of pediatric CD patients moved significantly further from the centroid of that of healthy controls one week after EEN treatment. However, at the end of their study, the gut microbiota composition of the good responders (but not the nonresponders) became closer to the centroid of healthy controls [20]. In other studies, while the gut microbiota diversity of the pediatric CD patients was not changed during EEN treatment, the relative abundance of Gram-positive bacteria (within the phylum Firmicutes) was increased and the relative abundance of the genera from the phylum Proteobacteria was decreased [21, 22]. Furthermore, Kaakoush et al. [23] have studied the alterations of the fecal microbiota in CD patients under EEN treatment. They found that CD remission occurred when the number of OTUs was decreased during EEN treatment; however, after the completion of EEN treatment, the OTUs increased and CD relapse simultaneously appeared [23]. These findings indicate that alterations of the gut microbiota are closely associated with the activity of CD.

Although variations of the gut microbiota diversity are not consistent in different studies, the microbiota composition was significantly changed during EEN treatment. Future studies should focus on the potential role of the gut microbiota composition in EEN-induced pediatric CD remission.

## 3. The Change of Immune Responses during EEN

The gut microbiota plays key roles in the development and maintenance of immune homeostasis. This can be firmly supported by the defective immune system in germ-free mice and the common gut microbiota dysbiosis in many autoimmune diseases [24, 25]. Body immune homeostasis is partly dependent on the balance of two subsets of CD4^+^ T cells: effector CD4^+^ T cells and regulatory CD4^+^ T cells (Treg cells). The function of the effector arm of the immune system is to recognize and eliminate pathogens. On the contrary, the purpose of the regulatory arm of the immune system, including Tregs that can produce the anti-inflammatory cytokine interleukin-10 (IL-10), is to suppress inflammation and to promote immunologic tolerance [26]. Of note, the gut microbiota can modulate the effector/regulatory T cell balance and subsequently promote immune homeostasis [27, 28]. Some species of Clostridia and* Bacteroides fragilis* have been found to stimulate the differentiation of colonic Treg cells, then induce the secretion of IL-10, and subsequently mitigate pathological inflammation and maintain intestinal health [29–31]. Guinet-Charpentier et al. [21] have found that, during EEN treatment, the fecal calprotectin levels and Harvey-Bradshaw scores of children with CD were negatively correlated to fecal Actinobacteria and Clostridia. In the same study, interestingly, fecal calprotectin was positively correlated to fecal *β*-Proteobacteria and the Harvey-Bradshaw score was positively correlated to *γ*-Proteobacteria [21]. The healthy intestinal microbiota is dominated by the phylum Firmicutes, especially the class Clostridia [32, 33].

The chronic relapse of CD is associated with intestinal immune imbalance, which is characterized by the dominance of pathogenic T helper 1 (T_H_1) and T_H_17 cells [34]. Takahashi et al. [35] have compared the microbial profiles between inactive CD patients and healthy controls and found that most of the CD patients experienced intestinal dysbiosis, in which the abundance of butyrate-producing bacterial species, such as* Faecalibacterium prausnitzii*, was decreased. Similarly, a decreased abundance of butyrate producers has been identified as the typical characteristic of intestinal dysbiosis in individuals that were at high risk for developing CD [36–38]. Short-chain fatty acids (SCFAs), including butyrate, acetate, and propionate, are produced by anaerobic commensal bacteria during the fermentation of nondigestible carbohydrates. SCFAs have been shown to be effective in promoting the secretion of mucin, increasing the number of colonic Treg cells, inducing the production of IL-10, and diminishing the production of proinflammatory mediators [39–45]. In other words, SCFAs are anti-inflammatory players in the maintenance of gut immune homeostasis. Dietary supplementation of SCFAs has been proposed as a potential method for treating chronic intestinal inflammation like IBD [46].

The microbial features in CD have been characterized as increased levels of proinflammatory members and decreased levels of anti-inflammatory mediators [15]. Breaking the established dysbiotic microbiota in CD may reduce intestinal inflammation. In a study by Schaubeck et al. [47], mice lacking tumor necrosis factor (TNF) that binds to adenosine-uridine-rich elements (ARE) (TNF^deltaARE^ mice), a CD-like ileitis mouse model, were housed under either conventional (CONV), specific pathogen-free (SPF) or germ-free (GF) conditions. Interestingly, the CONV-TNF^deltaARE^ mice developed ileal inflammation at week 18; however, the GF- TNF^deltaARE^ mice were free from inflammation [47]. In the same study, the transfer of the dysbiotic microbiota from SPF-TNF^deltaARE^ mice with severe ileitis to GF-TNF^deltaARE^ mice induced ileal inflammation; in contrast, the microbiota transplantation from healthy SPF-TNF^deltaARE^ mice (without ileitis) to GF-TNF^deltaARE^ mice induced no intestinal inflammation [47]. In addition, treatment with antibiotics altered the microbiota composition in inflamed CONV-TNF^deltaARE^ mice, included a reduction of Bacteroidetes, and significantly attenuated the ileitis; however, the microbiota composition returned to pretreatment levels 2 weeks later, which was followed by the subsequently increased severity of inflammation [47]. These findings suggest that the immunological condition is determined by the microbe-host interactions. Therefore, dysbiotic microbiota can disrupt the immune homeostasis and cause intestinal inflammation.

Microbiota variations may be one of the underlying mechanisms in EEN-induced pediatric CD remission because, during EEN treatment, the high remission rate of CD is always associated with extensive alterations of the gut microbiota composition [17, 48]. The disease activity and the intestinal inflammatory markers were significantly reduced at 3 weeks after EEN therapy. Of particular note, the EEN treatment increased the relative and absolute number of Treg cells in the peripheral blood, which could be explained by the significantly altered fecal bacterial communities [22]. Increased numbers of Gram-positive bacteria (within the phylum Firmicutes) and decreased numbers of Gram-negative bacteria (within the phylum Bacteroidetes) were also observed during EEN treatment [22].

## 4. Alterations of Microbial Metabolites after EEN Treatment

In order to study the impact of microbiota dysbiosis (in IBD) on bile acid metabolism and epithelial inflammation, Duboc et al. [49] compared the stool metabolic alterations between IBD patients and healthy controls. Increased levels of conjugated bile acids and 3-hydroxy-sulfated bile acids as well as decreased levels of secondary bile acids were observed in IBD patients. These results were explained by the impaired activity for deconjugating, transforming, and desulfating bile acids in the case of IBD. The secondary bile acids showed anti-inflammatory activities [49]. To determine the microbial metabolism alterations in the intestinal ecosystem of IBD patients, Morgan et al. [50] analyzed the microbiome from IBD patients and healthy controls. They observed decreased basic metabolism, increased oxidative stress pathway activity, and increased virulence and secretion pathways in the microbiome of ileal CD. Metagenomic analysis also has been conducted to compare the microbiota between healthy children and those with CD [19]. As a result, the children with CD had a reduced microbial diversity and a distinct microbiota composition. These results showed that, in CD, the increased modules were ubiquinone, lipopolysaccharide biosynthesis, and the twin-arginine translocation system. These were accompanied by a reduced frequency of key processes, such as the biosynthesis of fatty acid in the microbiome of CD patients [19].

Given the anti-inflammatory effect of SCFAs in maintaining intestinal homeostasis, one may expect that the production of SCFAs would increase during EEN treatment. Gerasimidis et al. [51] studied the alterations of the microbiota and the fecal metabolites during EEN treatment. However, in their study, decreased global microbial diversity,* F. prausnitzii*, and butyric acid levels as well as increased sulfide and fecal pH were observed during EEN treatment. Moreover, the SCFA content reached the lowest level at the end of EEN treatment in patients with juvenile idiopathic arthritis [52]. By using principal coordinate analysis, the authors showed that the samples collected after 5 weeks of EEN treatment clustered closely and had marked separation from the non-EEN samples, even though the samples were from two different treatment periods [52]. EEN induced a remarkable improvement in patients with juvenile idiopathic arthritis [52, 53]. The decreased SCFA production during EEN treatment could partially be explained by the low level or lack of fiber in the EEN formula.

Along with the decrease of SCFA production, the potentially toxic metabolites in CD patients, such as 1-propanol, 1-butanol, and the methyl and ethyl esters of SCFAs were also significantly decreased [54]. These findings imply that the overall alterations of the microbiota composition and their metabolites play an important role in inducing remission in the corresponding diseases during EEN treatment.

## 5. Role of the Microbiota in Determining Intestinal Immunologic Homeostasis

A healthy gut microbiota maintains the balance of carbohydrate metabolism, vitamin biosynthesis, and fatty acid synthesis, subsequently promoting the nutritional status, immune balance, and healthy physiological functions [55]. In other words, the gut microbiota composition is directly correlated to the phenotypes in the host (e.g., metabolic phenotypes and immunological phenotypes) [56, 57]. For example, in a mouse mutant model, an alteration in the microbiota composition was found to be associated with the host metabolic changes. Of particular interest, when the microbiome of those mice was transferred to wild-type mice, the same phenotype was reproduced [58–60]. Gut microbiota dysbiosis has been proposed as a major player in the progression of type 1 diabetes, since both prediabetes and diabetes patients had a distinct microbiota when compared with healthy individuals [61]. Alterations of microbiota composition prior to the initiation of type 1 diabetes also have been found in animal models [62]. In a mouse model, antibiotics reshaped the composition of the microbiota and increased the incidence of type 1 diabetes [63]. Taken together, the alterations of microbiota compositions can influence the immunological reaction.

The human intestine holds a high diversity of microbiota, which is predominated by Firmicutes, namely, Clostridia [32]. Firmicutes improve gut immune homeostasis by balancing the effector/regulatory T cell axis [64–66]. Basically, the gut microbiota dysbiosis in CD patients is shown as a disrupted balance between Firmicutes and Proteobacteria. The domination of Proteobacteria increases gut proinflammatory activities by inducing the production of effector CD4^+^ T cells [15, 67].

In a study by Dunn et al. [68], ten pediatric CD patients and five healthy controls were subjected to EEN treatment for 12 weeks. Nine out of these ten patients achieved CD remission at the end of EEN therapy. In addition, five out of these nine CD remission patients had sustained remission (SR) for at least 6 months; however, the other four patients experienced CD relapse soon after restarting a normal diet [68]. The 16S rRNA sequencing results showed that the species richness was lower in the CD patients, compared to the healthy controls. The lowest species richness was observed in the nonsustained remission (non-SR) patients. Moreover, the microbiota of the SR patients had a similar relative abundance of taxa, including the dominance of the phylum Firmicutes, like the healthy controls. However, in the non-SR patients, the proportion of the phylum Proteobacteria was much higher than those of the healthy controls and SR patients. Notably, the structure of the microbial community has been identified as a predictor for the outcome of EEN treatment in these patients [68].

EEN treatment has been reported to reduce the microbiota diversity of CD patients. Briefly, during EEN, the abundance of proinflammatory bacteria (such as Proteobacteria species) is decreased, and the abundance of anti-inflammatory bacteria is either decreased or increased [19, 21, 22, 69]. In other words, EEN treatment can induce a composition type that increases immunologic tolerance.

Firmicutes play an important role in maintaining gut microbial diversity. When dysbiosis occurs, the diversity is reduced, accompanied by a decrease of the relative abundance of Firmicutes as well as an increase of the relative abundance of Proteobacteria, especially Enterobacteriaceae [32, 70, 71]. The remission of CD occurs with an increase of microbial diversity, more specifically, an increase of the Firmicutes species and a decrease of the Proteobacteria species [10]. Microbial diversity is directly correlated to human health. An increased microbiota diversity represents an enhanced ability to resist intestinal inflammation [72]. From the ecosystematic perspective, the diversity determines the stability and function of an ecosystem. An ecosystem with a higher diversity is stronger than that with a lower diversity in resisting disturbance [73]. Similarly, according to the concept of “Network of Microbiota,” a higher diversity means more competition between members in the microbiota. This competitive relationship increases the stability of the ecosystem [74]. Diversity seems to be associated with a negative correlation present between Firmicutes and Proteobacteria species. Whether this correlation exists to maintain the microbiota composition stability and gut homeostasis has yet to be determined. If so, monitoring these species presents a promising avenue in clinical practice to maintain human health.

## 6. Conclusion

The microbiota composition, which is closely associated with microbial diversity and metabolic function, influences the immune homeostasis and the sensitivity to different diseases. Further investigation of the effects of the microbiota composition and the corresponding metabolic products on human immune homeostasis and disease development would offer us new ideas for therapy.

## Abbreviations

EEN: Exclusive enteral nutrition
CD: Crohn's disease
IBD: Inflammatory bowel disease
OUTs: Operational taxonomic units
Treg cells: Regulatory CD4^+^ T cells
IL-10: Interleukin-10
T_H_1 cells: T helper 1 cells
SCFAs: Short-chain fatty acids
TNF^deltaARE^ mice: Mice lacking tumor necrosis factor (TNF) that binds to adenosine-uridine-rich elements (ARE)
CONV: Conventional
SPF: Specific pathogen-free
GF: Germ-free
SR: Sustained remission
non-SR: Nonsustained remission.
